# The TF-miRNA Coregulation Network in Oral Lichen Planus

**DOI:** 10.1155/2015/731264

**Published:** 2015-05-03

**Authors:** Yu-Ling Zuo, Di-Ping Gong, Bi-Ze Li, Juan Zhao, Ling-Yue Zhou, Fang-Yang Shao, Zhao Jin, Yuan He

**Affiliations:** ^1^Teaching Hospital of Chengdu University of Traditional Chinese Medicine, Shierqiao Road 39, Chengdu, Sichuan 610072, China; ^2^Laboratory of Oral Biomedical Science and Translational Medicine, School of Stomatology, Tongji University, Middle Yanchang Road 399, Shanghai 200072, China; ^3^Preclinical Medicine College, Chengdu University of Traditional Chinese Medicine, Shierqiao Road 37, Chengdu, Sichuan 610075, China; ^4^Acupuncture and Tuina College, Chengdu University of Traditional Chinese Medicine, Shierqiao Road 37, Chengdu, Sichuan 610075, China; ^5^College of Basic Medicine, Chengdu University of Traditional Chinese Medicine, Shierqiao Road 37, Chengdu, Sichuan 610075, China

## Abstract

Oral lichen planus (OLP) is a chronic inflammatory disease that affects oral mucosa, some of which may finally develop into oral squamous cell carcinoma. Therefore, pinpointing the molecular mechanisms underlying the pathogenesis of OLP is important to develop efficient treatments for OLP. Recently, the accumulation of the large amount of omics data, especially transcriptome data, provides opportunities to investigate OLPs from a systematic perspective. In this paper, assuming that the OLP associated genes have functional relationships, we present a new approach to identify OLP related gene modules from gene regulatory networks. In particular, we find that the gene modules regulated by both transcription factors (TFs) and microRNAs (miRNAs) play important roles in the pathogenesis of OLP and many genes in the modules have been reported to be related to OLP in the literature.

## 1. Introduction

Oral lichen planus (OLP) is a chronic inflammatory disease that acts on mucous membranes inside the mouth and causes bilateral white lacy patches or plaques on the buccal mucosa, tongue, and gingivae [1]. It is found that OLP affects 0.5% to 2% of the adult population, especially the adults over 40 years old, where OLP tends to affect women rather than men with a ratio about 1.4 : 1 [2, 3]. Compared with cutaneous lichen planus, oral lichen planus lesions are more difficult to be treated with frequent recurrence. Furthermore, OLP may be at risk of developing into oral cancer as the result of carcinogenic exposures, where the erosive OLP lesions might be more sensitive to carcinogens than normal oral mucosa [1]. Currently, oral cavity cancer has become one of the 10 most frequently diagnosed cancers with increasing mortality in East Europe [4, 5]. However, the pathogenesis of OLP and how it is developed into oral cancer is still unclear [6, 7]. Therefore, it is extremely urgent to pinpoint the molecular mechanisms underlying the pathogenesis of OLP so that accurate diagnosis can be made and effective therapies can be developed.

Recently, the accumulation of large amount of omics data, especially transcriptome data, provides opportunities to identify the molecules related to diseases. Accordingly, many works have investigated OLPs with transcriptome data. For example, the genes CCR5, CD14, and beta-catenin have been identified to play important roles in the pathogenesis of OLP [8, 9]. Moreover, Tao et al. identified some genes that are differentially expressed in OLPs, such as FOXP3, ANGPT1, and MMP1, and these genes may be related to the development of OLP [10]. In general, the above-mentioned studies assume those differentially expressed genes between OLPs and controls are related to OLP. However, the differentially expressed genes are usually treated independently, which is actually not the case. It has been found that complex diseases, for example, cancers, happen due to the dysregulation of functional gene sets or molecular pathways [11, 12]. In other words, the genes involved in the same disease tend to have functional relationships. Therefore, it is necessary to investigate disease related genes from a systematic perspective. Except for the above-mentioned genes, some small noncoding RNAs, that is, microRNAs (miRNAs), were found to play important roles in cancer by targeting oncogenes or tumor suppressor genes [13]. For example, mir-21 was found to be overexpressed in several tumor types [14], and let-7 inhibits lung tumorigenesis by repressing the expression of the RAS oncogene [15]. More recently, Gassling et al. found that the dysregulation of some miRNAs has important pathophysiological impacts on OLP [16]. For instance, mir-21, mir-181b, and mir-345 were found to be upregulated in OLPs and have critical roles in the malignant transformation of OLP to oral cancer.

In this work, we present a novel approach to identify gene modules that may play important roles in the pathogenesis of OLP by assuming that OLP is caused due to the dysregulation of certain gene modules. Furthermore, based on the gene modules as well as their transcription factor and miRNA regulators, we construct a TF-miRNA coregulation network. By investigating the genes and their regulators in the coregulation network, we find that some of them have already been reported to be related to OLP or oral cancer, indicating the important roles of the regulation network in OLP. In addition, we notice that the genes involved in the regulation network can serve as disease associated pattern and separate OLPs from controls very well, which is also validated by another independent real dataset, demonstrating the potential of the gene modules we identified as disease associated pattern and therapeutic targets.

## 2. Materials and Methods

### 2.1. Gene and miRNA Expression Data

The matched gene and miRNA expression data were obtained from the Gene Expression Omnibus (GEO) database [17]. Both mRNA (accession number: GSE38616) and miRNA (accession number: GSE38615) expression profiles were measured in 7 healthy individuals and 7 oral lichen planus patients [16]. To further validate the genes identified from the above datasets, another gene expression dataset (accession number: GSE52130) was retrieved from GEO, which was originally measured in 14 oral samples and 9 genital epithelium samples. Here, the gene expression profiles from the 14 oral samples consist of 7 normal oral samples and 7 oral lichen planus samples were kept for validation. All the three expression datasets have been preprocessed and normalized when we downloaded them, and the data were used in later sections without further preprocessing.

### 2.2. Identification of Differentially Expressed Genes and miRNAs

In general, the genes that are differentially expressed between diseases and controls are related to diseases to some extent. In this work, the genes that are differentially expressed between OLPs and controls were detected with Student's *t*-test. The genes with *P* values less than 0.05 were regarded to be differentially expressed genes (DEGs), and the same for miRNAs. Consequently, 2587 differentially expressed genes (GSE38616) and 90 differentially expressed miRNAs (GSE38615) were obtained for further analysis.

### 2.3. Identification of Network Modules Associated with OLP

A gene coexpression network was constructed for OLPs based on their corresponding gene expression data, where one gene was linked to another if their coexpression measured with Pearson's correlation coefficient was significantly high (*P* value cutoff of 0.05) and the weights accompanying the edges were their corresponding correlation coefficients. Subsequently, network modules that consist of densely connected genes were detected with ClusterONE [18], and 154 network modules were detected here. Furthermore, after investigating the network modules, we merged two modules if more than one-third of genes from the smaller module occur in the larger one. As a result, 125 modules were kept for further analysis. For each network module, it will be regarded to be related to OLP if the module is enriched with the above identified differentially expressed genes, where the enrichment analysis was performed with Fisher's exact test (*P* value cutoff of 0.01).

### 2.4. miRNA-Gene Regulations

The target genes of miRNAs were collected from both predictions and experimentally determined ones. For the predictions, several tools, including PicTar [19], miRanda [20], MicroT [21], and TargetScan [22], were employed to predict the target genes of miRNAs. Specifically, we picked up the interactions between genes and miRNAs predicted by at least two tools to avoid false positives. Moreover, the target information of miRNAs deposited in Tarbase [23] was obtained and merged with the predictions, where all the miRNA-gene interactions from Tarbase have been experimentally validated.

### 2.5. The TF-miRNA Coregulation Network in OLP

After obtaining the network modules, we first checked which miRNAs may regulate the network modules. Given a network module and a miRNA, the miRNA will regulate the module if its target genes are enriched in the modules with Fisher's exact test (*P* value cutoff of 0.01). In particular, we only considered the differentially expressed miRNAs (DemiRs) here since these DemiRs are more likely related to OLP. Furthermore, given a network module, the transcription factors (TFs) that possibly regulate the modules were identified if these TFs belong to the module and coexpress with other genes in the module. Note that here we suppose the TFs that coexpress with other genes in the module will regulate the genes within the module. Consequently, we detected 6 network modules that are coregulated by TFs and miRNAs, where the modules are enriched with DEGs. We assumed that the TF-miRNA coregulation network which consists of the 6 modules plays important roles in the development of OLP.

## 3. Results and Discussion

**Figure 1:**
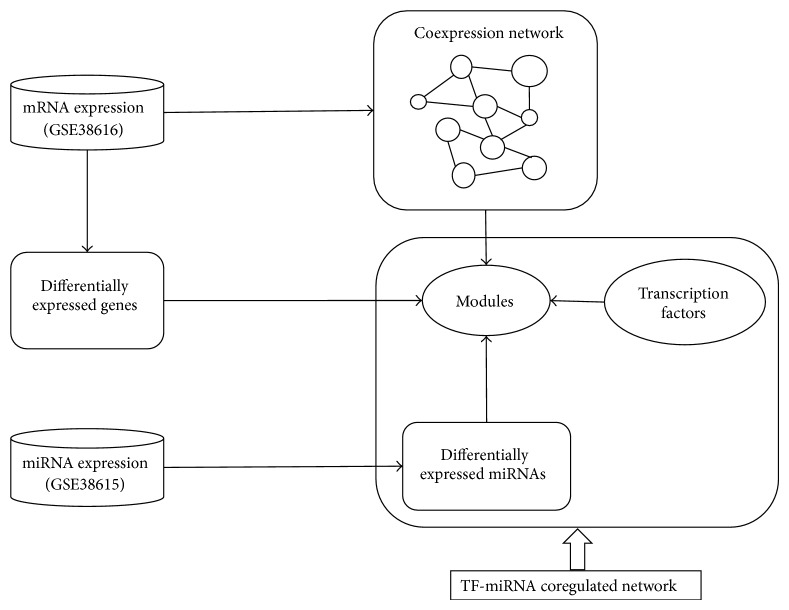
Schematic illustration of the pipeline to detect the TF-miRNA coregulation networks in oral lichen planus.

**Figure 2:**
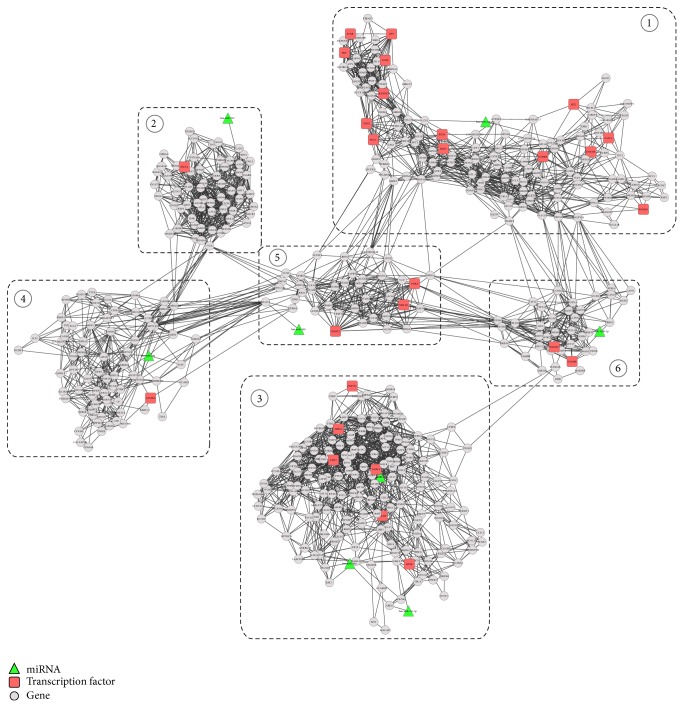
The TF-miRNA coregulation network with 6 modules, where the green nodes represent differentially expressed miRNAs, red nodes denote the transcription factors, and gray nodes represent genes in modules, respectively.

Figure 1 depicts the flowchart of our proposed approach for identifying the TF-miRNA coregulation network in OLP, and we applied it to a real dataset which contains 7 healthy individuals and 7 OLP patients, where the dataset contains the matched gene (GSE38616) and miRNA (GSE38615) expression profiles. From the dataset, we detected 2587 differentially expressed genes (DEGs) and 90 differentially expressed miRNAs (DemiRs). We further constructed a gene coexpression network and identified modules that were coregulated by miRNAs and TFs (Figure 2).  Table 1 lists the detailed information about the 6 network modules as well as their regulators.

### 3.1. The Network Modules Are Enriched with Oral Lichen Planus Related Genes

After getting the 6 network modules, we first investigated the genes in each module. By querying the PubMed, we found that 59 out of the 497 genes belonging to the 6 modules have been reported to be relevant to oral cancer. Here, for each network module, we gave examples about genes that have been reported to be related to OLP or oral cancer in the literature (Table 2). For example, it was reported that the gene KRT18 was related to tumor differentiation and metastasis and plays important roles in the malignant transformation of OLP to oral squamous cell carcinoma [24]. Another gene PTEN was reported to be downregulated in oral squamous cells, which in turn downregulates the expression of cyclin D1 and leads to the suppression of cell growth, indicating that targeting PTEN may help treat oral cancer [25]. Moreover, IGF1R has been reported to control cell proliferation of oral cancer [26, 27]. The overlap between known oral cancer associated genes and our identified module genes indicates that the genes belonging to these modules are related to OLP as well as its development to oral cancer.

Next, we investigated the functions of the network module genes. For each module, functional and pathway enrichment analyses were performed with DAVID [28], and the detailed results can be found in Table 3, where only the processes we thought related to OLP were listed for clarification. From the analysis, we can see the biological processes in which the modules involved are related to the initiation and development of OLP. For example, it was reported recently that the pathogenesis of OLP was associated with some systemic diseases that can cause midbrain injuries [29]. The inhibition of phospholipase A2 activity that is associated with numerous inflammatory processes was found to be related to the mechanism of OLP [30]. The sensory perception, such as anxiety and tension, has been reported to be an important factor in the development of OLP [31]. It was reported that oral lichen planus can be caused by a variety of stimuli and the preservation of keratin in oral mucosa was an efficient way for the treatment of the disease [32]. Compared with normal controls, the OLPs tend to have increased angiogenesis, indicating OLP is associated with the induction of aberrant angiogenesis [7]. In addition, the symptoms of OLP are always accompanied by compromised wound healing [33], and the epidermal growth factor receptors were found to be significantly higher in OLPs [34].

Except for biological processes, functional enrichments analysis implies that some molecular functions, such as cytosol, steroid hydroxylase activity, and oxidoreductase activity, also have important impacts on OLPs. Considering that OLP is often treated with steroids and vitamin A analogues [35], it is not surprising that steroid hormone receptor activity, retinoid metabolic process, and vitamin A metabolic process are enriched in our identified modules. Moreover, the metabolism of xenobiotics by cytochrome P450 has been reported to result in the oral and pharyngeal cancers [36]. In addition, it was found that the metallic ion content can increase the damage to the oral mucosa cells [37], which is consistent with the enrichment of the iron ion homeostasis and binding.

From the analysis of the genes belonging to our identified modules, we can see that these modules are indeed related to the development of OLP. In addition, we identified some important biological processes that have important roles in the development of OLP, such as the metabolism of xenobiotics by cytochrome P450 and vitamin A metabolic process. The detailed information about the biological processes in which the TFs and miRNAs are involved can be found in Supplementary Table I available online at http://dx.doi.org/10.1155/2015/731264.

### 3.2. miRNAs Regulators of Network Modules Are Associated with OLP

In the TF-miRNA regulation network, there are in total 8 miRNAs, which were picked up from the 90 differentially expressed miRNAs. Among the 8 miRNAs, some of them have been reported to be related to OLP or oral cancer in the literature. For example, miR-26b was found to be significantly low expressed in OLP lesions compared with controls [38], miR-29a was remarkably differentially expressed in the oral squamous cell carcinoma metastasis [39], and miR-628 was able to discriminate hand-foot-mouth diseases from healthy controls [40]. According to the Human microRNA Disease Database (HMDD) [41], a manually curated disease-miRNA association database, mir-146b was reported to be associated with diverse neoplasms including oral cancer. In addition, two of the 8 DemiRs, that is, hsa-miR-146b-5p and hsa-miR-26b, have been reported previously to be related to OLP [16].

**Figure 3:**
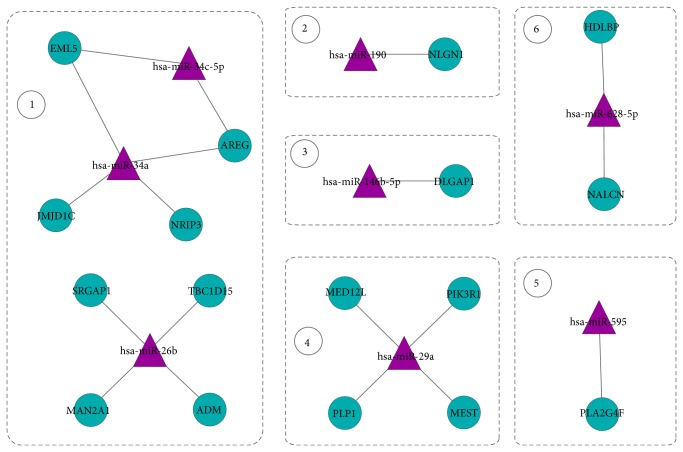
The regulatory relationship between miRNA and their targets in our regulatory network.

Furthermore, we derived the interactions between miRNAs and target genes from the 6 modules in TF-miRNA coregulation network.  Figure 3 shows the regulation network composed of miRNAs and target genes. By investigating the expressions of miRNAs and their target genes, we noticed that the expressions of 4 miRNAs, hsa-miR-190, hsa-miR-146b-5p, hsa-miR-29a, and hsa-miR-595, were negatively correlated with that of their target genes, which is consistent with the observation that miRNAs generally repress the expression of their target genes. Interestingly, these four miRNAs were highly expressed in OLP while their target genes were downregulated. The detailed information about the expression levels of miRNAs and their target genes in modules can be found in Supplementary Table II.

### 3.3. Transcription Factors Regulating the Network Modules Are Associated with OLP

We also investigated the 27 transcription factors involved in the TF-miRNA coregulation network. By querying the PubMed, some TFs were found already reported to be related to OLP or oral cancer. For example, in module 3, the transcription factor HIF1A is a master transcriptional regulator of the adaptive response to hypoxia. It was found that RTP801 and VEGF, the target genes of HIF1A, were significantly low expressed in OLPs [42]. The transcription factor LIN28A from module 4 has been reported to regulate cancer stem cell-like properties and can act as an appropriate target for oral squamous cell carcinoma treatment [43]. In module 1, AX6 was found to regulate the proliferation and apoptosis processes in human retinoblastoma cells [44]. In module 2, ASCL4 was found to be essential for the determination of cell fate as well as the development and differentiation of numerous tissues [45].

In addition, we investigated the top 25 biological processes regulated by these TFs as shown in Supplementary Figure 1, where the percentage denotes the fraction of all TFs from the TF-miRNA coregulation network that were involved in the corresponding process. Consistent with the above observations, the TFs identified here are involved in a lot of OLP related processes, such as cell differentiation, Notch signaling pathway, steroid hormone mediated signaling pathway, and wound healing.

The analysis of TFs involved in TF-miRNA coregulation network indicates that these TFs regulate OLP related biological processes and play important roles in promoting the progression and development of OLP.

## 4. Conclusion

The potential malignant transformation of oral lichen planus (OLP) to oral cancer makes it demanding to understand the pathogenesis of this disease. In this paper, we introduced a novel approach to identify the TF-miRNA coregulation network that plays important roles in OLP. Unlike traditional approaches, the regulatory circuit we detected here provides new insights into observing disease associated patterns. The overlap between known OLP associated genes and our identified module genes implies that these gene modules are significantly related to OLP. The discriminative capacity of these modules in separating OLPs from controls confirms again the important roles of these modules in OLP and their potential as disease associated pattern. In addition, the regulators of these gene modules, including transcription factors and miRNAs, were also found to play important roles in the manifestation and progression of OLP, indicating their potential as new therapeutic targets when treating OLPs.

## Supplementary Material

Figure S1: The distribution of biological processes regulated by transcription factors, where the percentage denotes the fraction of TFs that regulate the corresponding process.
Supplementary Table I: The detailed information about the biological processes in which the TFs and miRNAs are involved for 6 modules in TF-miRNA coregulation network.
Supplementary Table II:We derived the interactions between miRNAs and target genes from the 6 modules in TF-miRNA coregulation network. By investigating the expressions of miRNAs and their target genes, we noticed that the expressions of 4 miRNAs, hsa-miR-190, hsa-miR-146b-5p, hsa-miR-29a, and hsa-miR-595, were negatively correlated with that of their target genes.

## Figures and Tables

**Table 1:** The detailed information about the 6 network modules as well as their TF and miRNA regulators.

Module	#Nodes	#Edges	miRNAs	Transcription factors

Module 1	134	952	hsa-miR-628-5p	SALL2, PEG3, ZNF865, HES3, ZNF283, ZIM2, FOSB, PAX6, SIX2, ZNF616, KDM5D, SRY, RFX4, and ZFY
Module 2	62	594	hsa-miR-595	ASCL4
Module 3	160	1301	hsa-miR-34c-5p hsa-miR-34a hsa-miR-26b	ELK3, RFX8, MIXL1, HIF1A, and ZNF552
Module 4	80	405	hsa-miR-29a	LIN28A
Module 5	55	335	hsa-miR-190	THRA, NR1D1, and NR1D2
Module 6	43	241	hsa-miR-146b-5p	ZNF626

**Table 2:** Examples of genes from each network module that have been reported to be related to OLP or oral cancer.

Network module	Gene symbol	PubMed IDs

Module 1	KRT18	19575986; 22677743; 7527618;
	SHH	11857543; 21945071; 21496886
	FOSB	19653276; 15926923

Module 2	MAGEA3	19187853; 12855658
	KRT1	20002980; 16334838; 10896780
	MAGEA6	18197853;

Module 3	HIF1A	19717330; 19449077; 18630523
	PTEN	17067457; 15453811; 15805158
	IGF1R	17786320; 23106397; 19584075

Module 4	CP	23812204; 19884712; 17066447
	ABCG2	18429968; 15801936; 15618737
	ALDH1A1	22725270; 22782852; 21441790

Module 5	IDH1	22385606; 21383741; 19378339
	CRABP2	19197536; 16568407; 11437413

Module 6	CD55	21545652; 17234541; 15668483
	FGFR4	18487077; 20127014; 23481570
	CYP3A5	16338276; 18628519; 1808564

**Table 3:** Functional enrichment analysis of genes in network modules.

Network module	Enriched functions related to OLP	*P* value (<0.05)
Module 1	GO:0030901~midbrain development	0.00337086
	GO:0016702~oxidoreductase activity, acting on single donors with incorporation of molecular oxygen, incorporation of two atoms of oxygen	0.00560592
	GO:0008285~negative regulation of cell proliferation	0.01400315
	GO:0007600~sensory perception	0.032006
	GO:0007435~salivary gland morphogenesis	0.0376765
	GO:0002052~positive regulation of neuroblast proliferation	0.04294326

Module 2	GO:0004623~phospholipase A2 activity	0.00233
	GO:0050877~neurological system process	0.00764739
	GO:0051606~detection of stimulus	0.04745557

Module 3	GO:0001525~angiogenesis	0.00589117
	GO:0005829~cytosol	0.00679876
	GO:0042060~wound healing	0.01651834
	GO:0045740~positive regulation of DNA replication	0.01978034
	GO:0007173~epidermal growth factor receptor signaling pathway	0.01978034
	GO:0015629~actin cytoskeleton	0.04636455

Module 4	GO:0006879~cellular iron ion homeostasis	0.00604241
	GO:0000041~transition metal ion transport	0.029349054
	GO:0055114~oxidation reduction	0.03217
	hsa00980:Metabolism of xenobiotics by cytochrome P450	0.03978611

Module 5	GO:0003707~steroid hormone receptor activity	0.00495459
	GO:0006766~vitamin metabolic process	0.01044366
	GO:0001523~retinoid metabolic process	0.04657368
	GO:0006776~vitamin A metabolic process	0.04657368

Module 6	GO:0008395~steroid hydroxylase activity	0.02851299
	GO:0005887~integral to plasma membrane	0.02900047
	GO:0008202~steroid metabolic process	0.04570667
