# Supplementary material for: The TF-miRNA Coregulation Network in Oral Lichen Planus
Source: Biomed Res Int. 2015 May 3;2015:731264. doi: 10.1155/2015/731264 (PMC4433662; doi:10.1155/2015/731264)
Supplement: Supplementary file 1 — Figure S1: The distribution of biological processes regulated by transcription factors, where the percentage denotes the fraction of TFs that regulate the corresponding process. Supplementary Table I: The detailed information about the biological processes in which the TFs and miRNAs are involved for 6 modules in TF-miRNA coregulation network. Supplementary Table II:We derived the interactions between miRNAs and target genes from the 6 modules in TF-miRNA coregulation network. By investigating the expressions of miRNAs and their target genes, we noticed that the expressions of 4 miRNAs, hsa-miR-190, hsa-miR-146b-5p, hsa-miR-29a, and hsa-miR-595, were negatively correlated with that of their target genes. [file 731264.f1.zip › 731264.f1/supplymentary_submit/Supplementary Figure.docx]

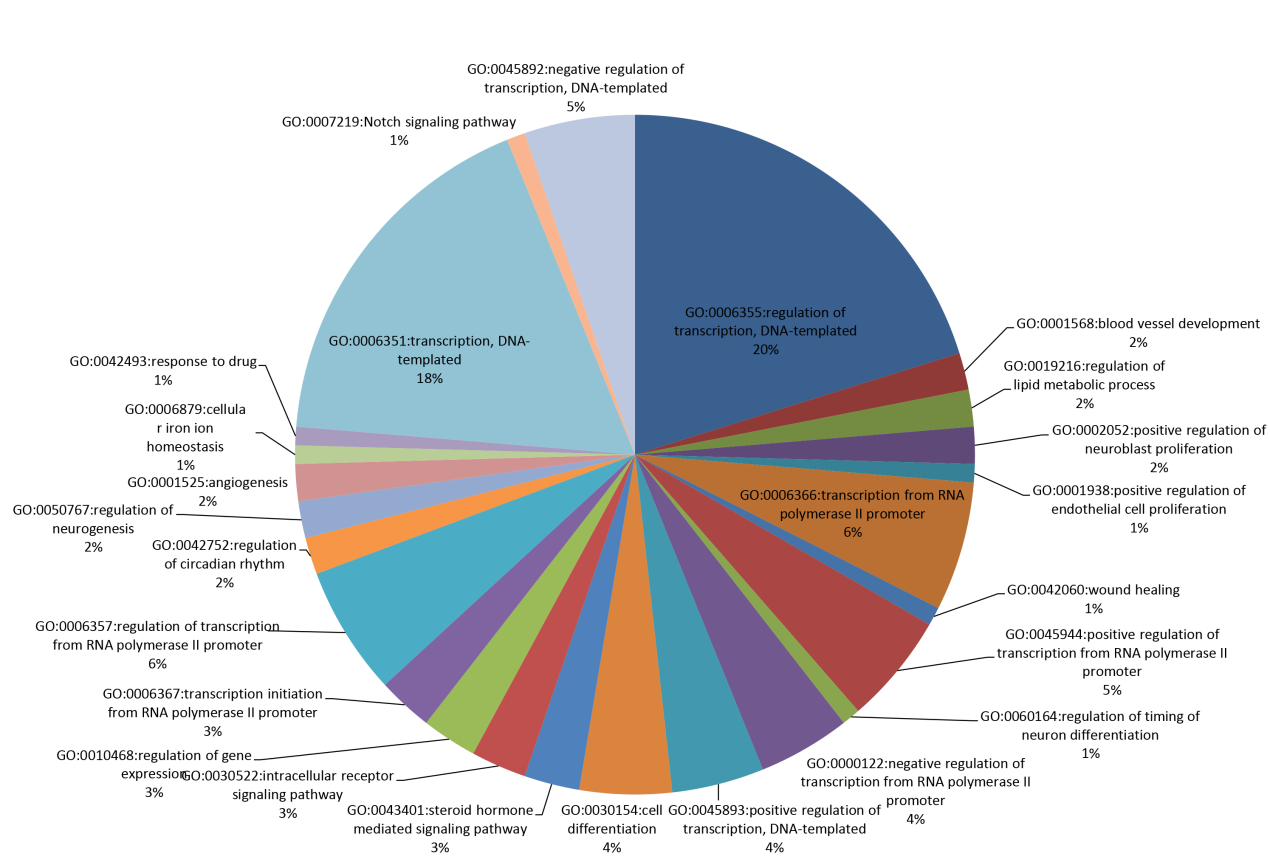


Fig S1. The distribution of biological processes regulated by transcription factors, where the percentage denotes the fraction of TFs that regulate the corresponding process.
